# Aurantio-Obtusin Attenuates Non-Alcoholic Fatty Liver Disease Through AMPK-Mediated Autophagy and Fatty Acid Oxidation Pathways

**DOI:** 10.3389/fphar.2021.826628

**Published:** 2022-01-11

**Authors:** Fei Zhou, Mingning Ding, Yiqing Gu, Guifang Fan, Chuanyang Liu, Yijie Li, Rong Sun, Jianzhi Wu, Jianchao Li, Xiaoyong Xue, Hongjuan Li, Xiaojiaoyang Li

**Affiliations:** ^1^ School of Life Sciences, Beijing University of Chinese Medicine, Beijing, China; ^2^ School of Chinese Materia Medica, Beijing University of Chinese Medicine, Beijing, China; ^3^ School of Traditional Chinese Medicine, Beijing University of Chinese Medicine, Beijing, China; ^4^ The Second Hospital of University, Jinan, China; ^5^ Advanced Medical Research Institute, Shandong University, Jinan, China; ^6^ Shandong University of Traditional Chinese Medicine, Jinan, China

**Keywords:** aurantio-obtusin, nonalcoholic fatty liver disease, autophagy, AMPK, PPARα, ACOX1

## Abstract

Nonalcoholic fatty liver disease (NAFLD), manifested as the aberrant accumulation of lipids in hepatocytes and inflammation, has become an important cause of advanced liver diseases and hepatic malignancies worldwide. However, no effective therapy has been approved yet. Aurantio-obtusin (AO) is a main bioactive compound isolated from *Cassia* semen that has been identified with multiple pharmacological activities, including improving adiposity and insulin resistance. However, the ameliorating effects of AO on diet-induced NAFLD and underlying mechanisms remained poorly elucidated. Our results demonstrated that AO significantly alleviated high-fat diet and glucose-fructose water (HFSW)-induced hepatic steatosis in mice and oleic acid and palmitic acid (OAPA)-induced lipid accumulation in hepatocytes. Remarkably, AO was found to distinctly promote autophagy flux and influence the degradation of lipid droplets by inducing AMPK phosphorylation. Additionally, the induction of AMPK triggered TFEB activation and promoted fatty acid oxidation (FAO) by activating PPARα and ACOX1 and decreasing the expression of genes involved in lipid biosynthesis. Meanwhile, the lipid-lowing effect of AO was significantly prevented by the pretreatment with inhibitors of autophagy, PPARα or ACOX1, respectively. Collectively, our study suggests that AO ameliorates hepatic steatosis *via* AMPK/autophagy- and AMPK/TFEB-mediated suppression of lipid accumulation, which opens new opportunities for pharmacological treatment of NAFLD and associated complications.

## Introduction

Nonalcoholic fatty liver disease (NAFLD), characterized by excess hepatic fat accumulation, inflammation and oxidative stress, has become a burgeoning worldwide epidemic liver disease, affecting approximately one-quarter of the entire global population (Diehl and Day, 2017). Moreover, NAFLD not only encompasses a cascade of conditions including nonalcoholic steatohepatitis (NASH), advanced liver fibrosis, cirrhosis and even hepatocellular carcinoma (HCC) but also confers a high risk for type 2 diabetes, cardiovascular complications and various extrahepatic malignancies (Dixon et al., 2001; Loomba et al., 2020). So far, the underlying mechanism driving NAFLD development remains obscure. An increasing number of studies have pointed out that the pathophysiology of NAFLD is complex and multifactorial. Progress over the last decade was substantial in the roles of lipotoxicity, oxidative stress, inflammation, genetics and metabolism in NAFLD pathogenesis (Marra and Svegliati-Baroni, 2018). Furthermore, gut microbiome and various dietary components as other gastrointestinal hits with proinflammatory potential have been demonstrated (Tilg et al., 2021). Among these pathogenic factors, lipotoxicity, resulting from excessive accumulation of harmful lipids and hepatocyte injury, has gained remarkable attention and represents a critical step in NAFLD progression (Marra and Svegliati-Baroni, 2018).

Autophagy is a genetically programmed and evolutionarily conserved self-eating catabolic process whereby injurious organelles and intracellular components are degraded within lysosome. Under pathological circumstances, autophagy plays a critical role in maintaining cellular and metabolic homeostasis by eliminating damaged organelles, misfolded proteins as well as accumulated lipid droplets (Singh et al., 2009). Recently, insufficient autophagy was regarded as the fundamental cellular defect in the pathogenesis of NAFLD. Notably, the dysregulation of hepatic autophagy has been implicated in NAFLD and NASH patients (Czaja, 2016). In experimental animals, the blockade of autophagy using selective inhibitors or specific siRNAs targeting autophagy-related genes like autophagy-related protein 5 (ATG5, *Atg5*) promoted the hepatic accumulation of lipid droplets and triglycerides (TG) and accelerated liver injury (Ni et al., 2012; Zhou et al., 2018). Furthermore, the enhancement of autophagy accelerated fatty acid oxidation (FAO), improved mitochondrial function and protected against palmitate lipotoxicity in murine hepatocytes (Mwangi et al., 2019). However, whether autophagy in NAFLD could be targetable by any pharmacological therapies remains unclear.

At present, therapeutic approaches of NAFLD mainly include dietary restrain or taking medicines inhibiting lipid biogenesis, removing excessive lipid droplets, and inhibiting inflammation or cell apoptosis in livers (Mao et al., 2016). Notably, the primary therapeutic strategy for NAFLD is targeting intrahepatic lipid accumulation, such as the agonists of FAO including peroxisome proliferator-activated receptor alpha (PPARα) agonists, acyl-coenzyme A oxidase 1 (Acox1) agonists and inhibitors of *de novo* lipogenesis including fatty acid synthase (FASN) inhibitors. Although most representative next-generation PPARα or pan-PPARs agonists like elafibranor and pioglitazone have been found to efficiently induce the resolution of NASH patients, most of them have been withdrawn from the market owing to severe side effects such as weight gain, bone fractures, peripheral edema and even congestive heart failure (Friedman et al., 2018; Boeckmans et al., 2019). Besides, a variety of synthetic inhibitors, including apoptosis signaling kinase 1 (ASK1) inhibitors, galectin three antagonists and nonsteroidal FXR agonists, have been reported to improve advanced NASH patients in clinical trials; however, their applications are still restricted due to unexpected adverse effects like pruritus (Sumida and Yoneda, 2018). Recently, autophagy represents a novel therapeutic target for NAFLD by clearing excessive lipid droplets and suppressing hepatic inflammation, while the unclear and double-edged effects of autophagy raise concerns as well (Mao et al., 2016). Despite these tough challenges, there is as yet no Food and Drug Administration (FDA)-approved pharmacotherapy, and it remains a pressing need to discover interventions to mitigate the risk of hepatic steatosis.

Unlike the above strategies that mainly focused on one single target, natural products derived from traditional Chinese medicine (TCM) with multiple-targeting and promising hepatoprotective effects represent the rich source of lead compounds for drug discovery against liver steatosis. Aurantio-obtusin (AO), the main characteristic bioactive ingredient isolated from *Cassiae* semen that has a long history of usage for thousands of years, exerts a broad spectrum of pharmacological activities, including anti-inflammatory, anti-allergic, anti-hypertensive, antioxidant and immunoregulatory effects (Kim et al., 2015; Kwon et al., 2018; Tejero et al., 2019). Recently, increasing studies have begun to focus on the unique effects of AO on improving metabolic diseases. Under the challenge of high fat and sweet sugar, AO was found to improve adiposity and insulin resistance by downregulating the expression of lipid metabolism-related genes and suppressing inflammatory cytokines expression in white adipose tissue (Guo et al., 2021). Although the above results pointed out the possibility of AO-induced hepatoprotective effects against liver steatosis, researchers temporarily focused on the anti-obesity effect of AO while paid less attention to its lipid-reducing effect and deeper mechanism involved in NAFLD. These insightful preliminary results and existing questions encourage us to further investigate the potential protective effects of AO on diet-induced hepatic steatosis.

In the current study, we investigated the therapeutic effects and underlying mechanisms of AO in a high-fat diet (HFD) and glucose-fructose water (HFSW)-induced NAFLD mouse model and oleic acid and palmitic acid (OAPA)-treated mouse primary hepatocytes (MPHs). AO markedly promoted autophagy flux, triggered transcription factor EB (TFEB) activation, subsequently inhibited lipid *de novo* synthesis and accelerated FAO in livers by inducing AMP-activated protein kinase (AMPK) phosphorylation, which were prevented by pretreatment with the inhibitors of autophagy, PPARα or ACOX1, respectively. Taken together, our findings provide experimental evidence supporting the possibility of developing AO-based medicines for the pharmacological treatment of NAFLD and associated metabolic disorders.

## Materials and Methods

### Materials

AO (PS0100-0250) was purchased from Push Bio-Technology (Chengdu, China). Oleic acid (C4977) was obtained from APExBIO (Houston, United States). Palmitic acid (P101061) was purchased from Aladdin (Shanghai, China). 3-methyladenine (3-MA) (S2767) and compound C (CC) (HY-13418A) were obtained from Selleck (Houston, United States). 10,12-Tricosadiynoic acid (TRCDA), glucose (B21882) and fructose (B21896) were purchased from Yuanye Bio-Technology (Shanghai, China). Williams’ Medium E with l-glutamine (W4125), collagenase from *Clostridium* histolyticum (C5138), collagen type I from rat tail (C3867), dexamethasone (D1756), 0.1% l-Thyroxine (T-073) and other cell culture material were purchased from Sigma (St. Louis, United States). Goat anti-mouse IgG (H + L) Highly Cross-Adsorbed secondary antibody (Alexa Fluor Plus 488) (Ul287767) was obtained from Thermo Fisher Scientific (Waltham, United States) and goat anti-rabbit IgG (H + L), F (ab')2 Fragment (Alexa Fluor 594 Conjugate) (8889S) was purchased from Cell Signaling Technology (Danvers, United States).

### Animal Study

C57BL/6J (8-week-old, male) mice were obtained from Vital River Laboratory Animal Technology (Beijing, China). Mice were kept under 12 h light-dark cycle at a consistent temperature (22 ± 2 C) with free access to water and standard chow. All animals were accepted 1 week of adaptive feeding before the experiment and sacrificed after 8-weeks treatment. For *in vivo* experiments, the doses of AO (5,10 and 15 mg/kg) were selected based on recent publications (Xu et al., 2019; Guo et al., 2021). In chronic HFSW experiment (n = 6), mice were divided into five groups: 1) control group (chow diet); 2) NAFLD model group [HFSW, Western diet-42% Kcal from fat and 0.2% cholesterol (TD.88137, Harlan Laboratories, Inc., Indianapolis, IN, United States) plus with a high sugar solution (d-fructose: 23.1 g/L and d-glucose: 18.9 g/L) in drinking water (Li et al., 2021)]; (3–5) HFSW diet with AO administration group. Mice in groups (2–5) were first fed with the HFSW diet for 4 weeks to induce liver steatosis followed by orally given different doses of AO (5, 10 and 15 mg/kg) or vehicle control for another 4 weeks. In the ACOX1 inhibitor experiment, mice were divided into five groups at random (n = 6): 1) control group; 2) HFSW diet group; 3) HFSW diet with AO administration group; 4) HFSW diet with TRCDA administration group; 5) HFSW diet with AO and TRCDA administration group. Mice from groups (4)–5) were intragastrically administrated with TRCDA (1 mg/kg) for 8 weeks and mice from groups (3)–5) orally administrated with AO (10 mg/kg) in the last 4 weeks. Body weight, food intake and water consumption were measured every 2 days. At the end of treatment, mice were anesthetized and shaved in the thorax and abdomen area for measuring body temperature using Tau two VPC thermal imaging camera (FLIR Systems, United States). After sacrificed, mice serum and liver tissues were collected for further experiments.

All animal studies and procedures were approved by the Institutional Animal Care and Use Committee of Beijing University of Chinese Medicine and were carried out in accordance with all guidelines.

### Measurement of Serum Alanine Transaminase and Aspartate Transaminase

Mice serum was collected after sacrificed. ALT assay kit (C009-2-1) and AST assay kit (C010-2-1) were obtained from Nanjing Jiancheng Bioengineering Institute (Nanjing, China) and used to measure serum ALT and AST levels according to the manufacturer’s instructions.

### Measurement of Total TG and Total Cholesterol

Mouse serum was collected by centrifugation and liver samples were homogenized by anhydrous ethanol for obtaining supernatant. Cell samples were lysed in RIPA lysis buffer and prepared the supernatant for following detection. TG and TC from different samples were measured using TG assay Kit (A110-1-1) and TC assay kit (A111-1-1) from Nanjing Jiancheng Bioengineering Institute (Nanjing, China).

### Histopathologic Analysis

Mice liver tissues were fixed with 4% formaldehyde for 7 days, dehydrated by ethyl alcohol, embedded by paraffin and cut into 5 μM sections. For hematoxylin and eosin (HE) staining, paraffin sections were dewaxed by xylene, rehydrated by ethyl alcohol, stained by hematoxylin and eosin and dehydrated by ethyl alcohol. The images of all sections were captured by Aperio Versa (Leica, Wetzlar, Germany).

### Oil-Red Staining

Oil-red staining was performed as previous established conditions (Li et al., 2021). Oil-red powder was first dissolved in isopropanol solution (0.5 g/ml). Freshly cut sections from frozen livers and hepatocytes were fixed in 4% formaldehyde, washed in PBS solution and subsequently stained by Oil-red solution for 30 min. The image was captured by Aperio Versa (Leica, Wetzlar, Germany).

### Isolation and Culture of Mouse Primary Hepatocytes

MPHs were isolated by a two-step collagenase perfusion method as previously described (Li et al., 2017). After seeded in 6-well plates or different dishes that pre-coated with collagen, MPHs were cultured in Williams’ Medium E containing with 0.1% dexamethasone and 0.1% l-Thyroxine for further experiments.

### Cell Treatment

For *in intro* experiments, the doses of AO were chosen according to our CCK8 data. For the time course experiment, MPHs were treated with DMSO or AO (25 μM) for 0.5, 1, 2, 4 and 6 h. For the dose course experiment with or without OAPA, MPHs were first treated with OAPA (OA: 250 μM, PA: 500 μM) or not and then treated with different dosages of AO (12.5, 25 and 50 μM) for 24 h. For inhibitor experiments with or without OAPA, MPHs were first treated CC (10 μM), 3-MA (5 mM), TRCDA (10 μM) or GW6771 (10 μM) for 1.5 h, respectively, then treated with OAPA or not and AO (25 μM) for another 2 h or 24 h.

### HepG2 Cells Transfection

HepG2 cells were obtained from ATCC and were seeded in a 6-well plate at 30×10^4^ cells/well for 24 h. After attachment, cells were cultured with complete medium (CM) (1% Penicillin-Streptomycin and 10% FBS) containing HBLV-mcherry-EGFP-LC3-PURO (MOI = 20) (HanBio Technology, China) and polybrene (5 μg/ml) for 8 h. After virus infection, cell culture medium was changed into fresh CM for 16 h and then replaced with selection medium (CM containing 2.5 μg/ml puromycin). After 1 week of selection, cells were treated with AO (25 μM) or DMSO for 3 and 5 h. Live cell images were captured by Olympus FV3000 confocal laser scanning microscopy (Tokyo, Japan).

### Quantitative Real-Time RT-PCR

Liver tissues were homogenized and lysed by TRIzol reagent. Total RNA from mouse livers were extracted by chloroform, isopropanol and 75% ethyl alcohol and dissolved in DNase/RNase-free water. Total RNA of cell samples was extracted with Fast Pure Cell/Tissue Total RNA isolation kit (RC101-01, Vazyme Biotech). The mRNA expression was detected by quantitative real-time PCR (qPCR) using AceQ universal SYBR qPCR Master Mix (Q511-02, Vazyme Biotech). Further inquiries of primers for qPCR can be directed to the corresponding author.

### Western Blot Analysis

Western blot analysis was carried out as previously described (Li et al., 2021). Briefly, liver tissues and cell samples were lysed in RIPA lysis buffer. The equivalent protein was prepared, separated with SDS-PAGE gel and successively incubated with relative primary antibodies at 4 C overnight. After incubated with secondary antibodies, all bands were developed using ECL western bolting reagents and imaged by ChemiDocTM Touch Imaging System (Bio-Rad, Hercules, CA). Detailed information of primary antibodies was provided in Supplementary Table S1.

### Statistical Analysis

All results were repeated at least three times and presented as mean ± SEM. One-way ANOVA was employed to compare the differences between multiple groups using GraphPad Prism 8 (Graph-Pad, San Diego, CA). *p* value ≤ 0.05 was considered statistically significant.

## Results

### AO Ameliorates Hepatic Steatosis in HFSW-Fed Mice

**FIGURE 1:**
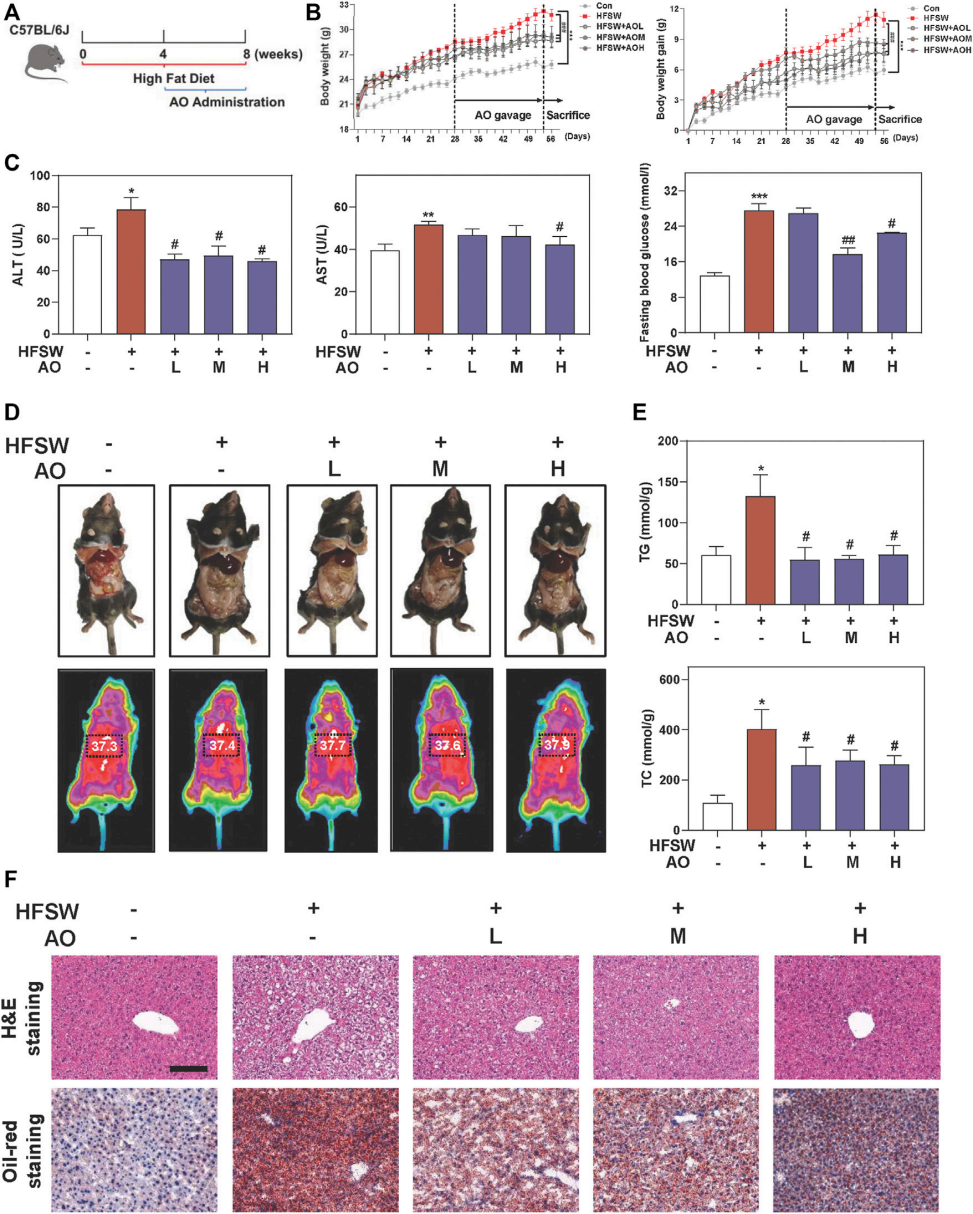
AO ameliorates hepatic steatosis in HFSW-fed mice. Mice were fed with chow diet or HFSW diet for 8 weeks and administered with different dosages (5, 10, and 15 mg/kg) of AO by gavage from Week 5 **(A)** Schematic diagram of *in vivo* experimental design **(B)** Body weight and Body weight gain **(C)** Levels of serum ALT and AST and fasting blood glucose **(D)** Representative images of epididymal fat and thoracoabdominal temperature **(E)** Hepatic TG and TC levels **(F)** Representative images of H&E and oil red O staining. Scale bar = 100 μm. Statistical significance: *p < 0.05, **p < 0.01, ***p < 0.001, compared with control group; ^#^ *p* < 0.05, ^##^ *p* < 0.01, compared with HFSW group (n = 6).

To identify the hepatoprotective effects and detailed mechanisms of AO on fatty liver, mice were first fed with an HFSW diet for 4 weeks to induce liver steatosis followed by administration with different doses of AO (5, 10 and 15 mg/kg) or vehicle control for additional 4 weeks with continuous HFSW diet (Figure 1A). The body weight and the intake of food and water were recorded every 2 days. As shown in Figure 1B and Supplementary Figure S1A, AO significantly reduced the HFSW-induced body weight and body weight gain without affecting the amount of average calorie and water intake. Moreover, analysis of the raw tissues of liver, spleen and adipose showed that AO slightly decreased the organ coefficients of spleen and adipose but had no obvious influence on livers (Supplementary Figure S1B–1D). Serum biochemistry assays then demonstrated that AO significantly decreased the serum ALT and AST levels in HFSW mice (Figure 1C, **left** and **middle panel**). As expected, HFSW also significantly increased the serum levels of blood glucose, which were markedly reversed by medium dose and high dose of AO (Figure 1C, **right panel**). More intuitively, we examined the accumulation of abdominal fat and measured the thoracoabdominal temperature and found that AO resulted in a less weight of white adipose tissue and a greater liver temperature than that in the HFSW group (Figure 1D), suggesting that AO improved metabolic syndrome of HFSW mice. As illustrated in Figure 1E and Supplementary Figure S1E, HFSW also significantly elevated the serum and hepatic lipid accumulation, as evidenced by increased levels of TG and TC. As expected, AO administration markedly reduced hepatic TG and TC as well as serum TG levels. In line with the above findings, histological examination depicted that AO significantly and dose-dependently reduced the HSFW-caused lipid droplet accumulation and extensive steatosis, as indicated by decreased number and size of lipid droplets in hepatocytes (Figure 1F and Supplementary Figure S1F). These results suggested that AO exerted beneficial effects on hepatic steatosis.

### AO Regulates Lipid Metabolism and Autophagy in HFSW-Fed Mice

**FIGURE 2:**
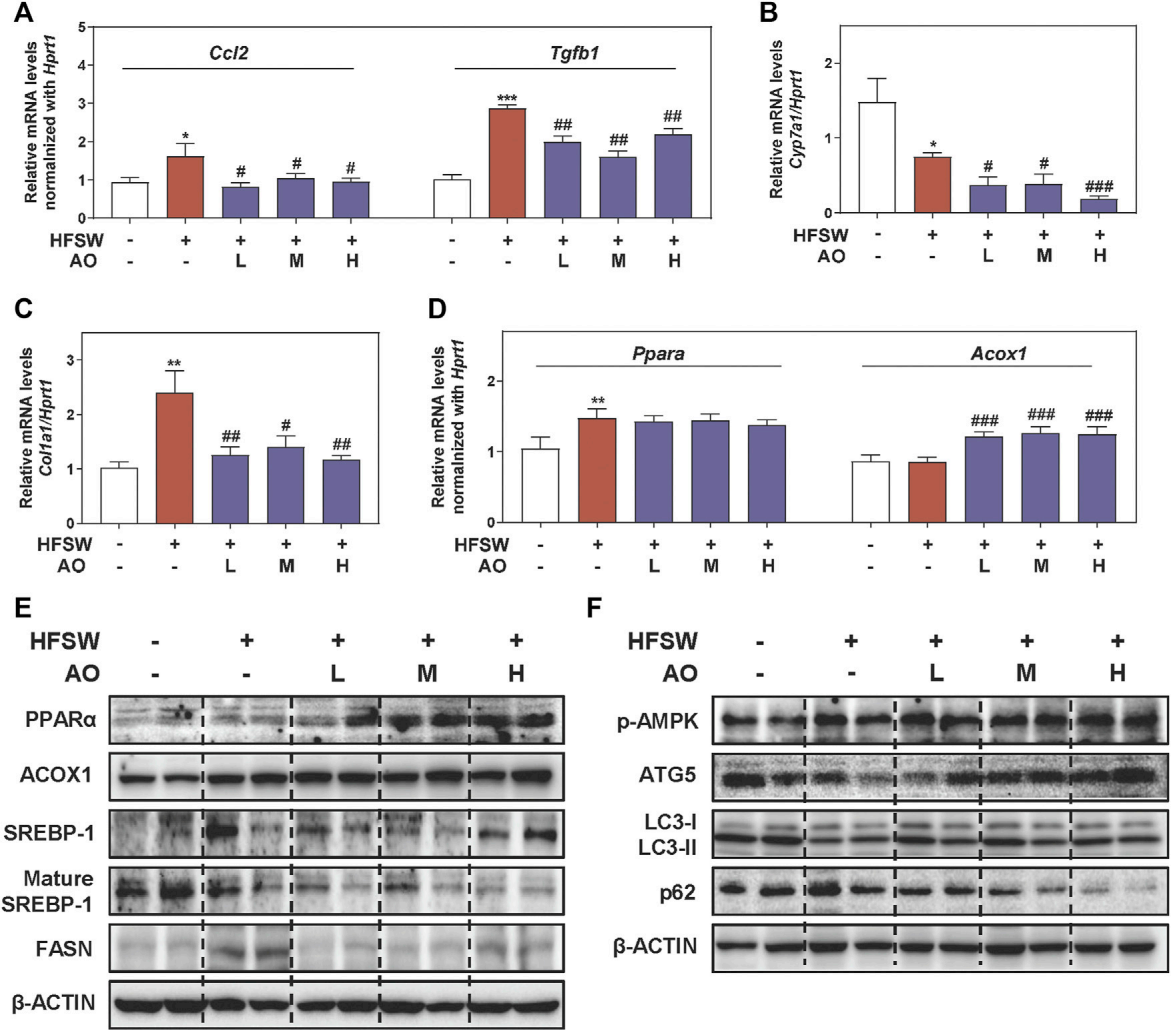
AO improves lipid metabolism and activates autophagy pathway in HFSW-fed mice. Mice were treated with the same method as described in Figure 1. Relative mRNA levels of **(A)** *Ccl2* and *Tgfb1*, **(B)** *Cyp7a1*, **(C)** *Col1a1* and **(D)** *Ppara* and *Acox1* in liver tissues were determined by qPCR and normalized using *Hprt1* as an internal control. The protein levels of **(E)** PPARα, ACOX1, SREBP-1, mature SREBP-1, FASN, **(F)** p-AMPK, ATG5, LC3-II/LC3-I and p62 in the liver were determined by western blot using β-ACTIN as a loading control. Statistical significance: *p < 0.05, **p < 0.01, ***p < 0.001, compared with control group; ^#^ *p* < 0.05, ^##^ *p* < 0.01, ^###^ *p* < 0.001, compared with HFSW group (n = 6).

Considering that inflammatory response toward hepatocyte damage is a decisive step in the development of NAFLD, we examined the levels of several major inflammatory cytokines and chemokines for immune cell recruitment. As shown in Figure 2A, HFSW significantly increased the levels of *Ccl2* and *Tgfb1* in livers, which were then markedly downregulated under AO administration. However, the effect of AO on HFSW-induced *Tnf* and *IL-6* was minimal (**data not shown**). Once out of control, persistent inflammation will disturb the bile acid homeostasis and lead to a cumulative result-liver fibrosis, which in turn, reinforces a vicious cycle of NAFLD damage (Chavez-Talavera et al., 2017). Cholesterol metabolism enzyme cholesterol 7-alpha-hydroxylase (CYP7A1, *Cyp7a1*), a primary rate-limiting enzyme that promotes the conversion of cholesterol into bile acids, was downregulated by HFSW probably due to a compensatory mechanism but was markedly reduced under all doses of AO (Figure 2B). Next, we measured the mRNA expression of genes involved in ECM accumulation and liver fibrosis like *Fn1* and collagen type I (COL1A1, *Col1a1*). Although the *Fn1* level was almost unchanged before or after AO treatment (Supplementary Figure S2A, **left panel**), the administration of AO significantly decreased the mRNA level of *Col1a1* upon HFSW feeding (Figure 2C). Subsequently, the most prominent genes involved in lipid synthesis and metabolism such as *Fasn* and key regulators of FAO (*Ppara* and *Acox1*) were also determined after AO treatment. As depicted in Figure 2D and Supplementary Figure S2A, **right panel**, AO didn’t affect the *Ppara* and *Fasn* levels caused by HFSW but significantly elevated the *Acox1* level in the fatty liver. We next analyzed representative proteins in pathways coordinating lipid homeostasis. PPARα contributes to the transcriptional regulation of essential genes involved in *de nova* fatty acid biosynthesis, including *Srebp1* and *Fasn* (Shen et al., 2020). As shown in Figure 2E and Supplementary Figure S2B, the protein levels of PPARα and ACOX1 were increased while FASN and the transformation of uncleaved SREBP-1 into mature SREBP-1 were decreased in AO-treated mice than their respective levels in HFSW mice, providing the potential explanation for the increased FAO and liver temperature after AO administration in Figure 1D. However, the expression of carnitine palmitoyl transferase 1alpha (CPT1α, *Cpt1a*), another important target responsible for mitochondrial oxidation, was almost unchanged after AO treatment (Supplementary Figure S2C). A series of other and our studies recently reported the important role of AMPK played in maintaining physiological functions and alleviating fatty liver, which might attribute to the regulation of autophagy and lipid metabolism (Zhao et al., 2020; Wu et al., 2021). As depicted in Figure 2F and Supplementary Figure S2D, the phosphorylation of AMPK was markedly increased after AO administration. We also observed that AO resulted in a significant increase of LC3-II/LC3-I and ATG5 levels and a decrease of p62 level in livers compared with the HFSW group. These findings together with those presented in Figure 1 supported that AO inhibited fatty acid synthesis, promoted FAO and activated AMPK signaling and autophagy in response to lipid challenge.

### AO Alleviates Lipid Accumulation in Fat-Overloaded MPHs

**FIGURE 3:**
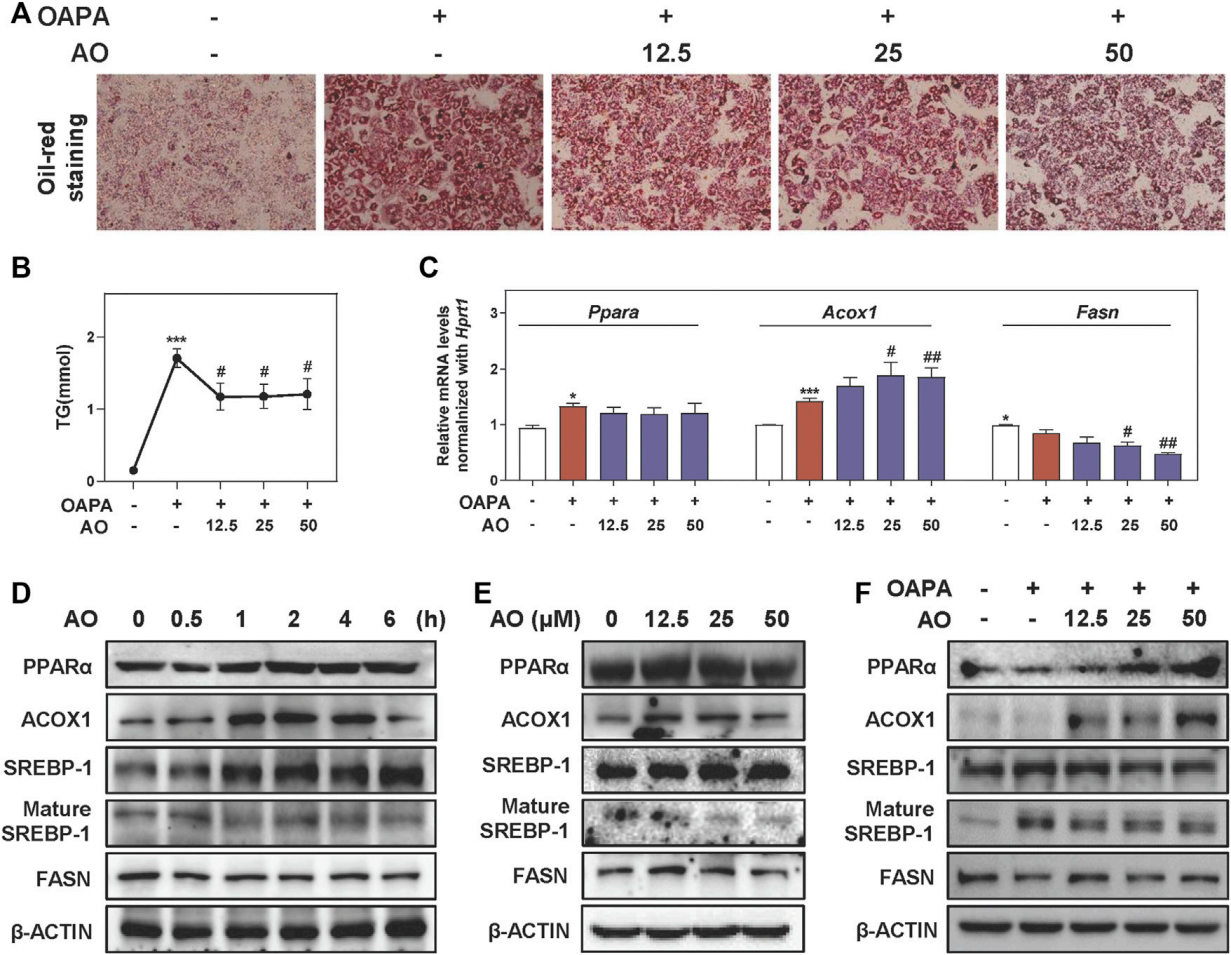
AO alleviates lipid accumulation in fat-overloaded MPHs. MPHs were incubated with OAPA (OA: 250 μM, PA: 500 μM) and treated with different dosages (12.5, 25 and 50 μM) of AO for 24 h **(A)** Representative images of oil red O staining of MPHs **(B)** TG levels of MPHs **(C)** Relative mRNA levels of *Ppara*, *Acox1* and *Fasn* in MPHs were determined by qPCR and normalized using *Hprt1* as an internal control **(D)** MPHs were treated with AO (25 μM) at different time points **(E** and **F)** MPHs were treated with different concentrations of AO for 24 h with or without OAPA **(D, E** and **F)** The protein levels of PPARα, ACOX1, SREBP-1, mature SREBP-1 and FASN in MPHs were determined by western blot using β-ACTIN as a loading control. Statistical significance: *p < 0.05, ***p < 0.001, compared with control group; ^#^ *p* < 0.05, ^##^ *p* < 0.01, compared with OAPA group (n = 3).

To further investigate lipid-lowering effects of AO on hepatocytes and gain detailed insight into the working mechanisms, we freshly isolated MPHs and further established an OAPA-induced steatosis *in vitro* model to mimic the *in vivo* environment. Our CCK-8 assay showed that AO had no obvious influence on MPH cell viability even at 200 μM (Supplementary Figure S3A). As shown in Figure 3A, the increased number and size of lipid droplets in hepatocytes were observed after the OAPA insult, which were distinctly and dose-dependently reversed by AO treatment (from 12.5 to 50 μM). Therefore, doses below 50 μM of AO were selected for the following *in vitro* assays. Next, the TG contents in MPHs were measured with 11.2 times higher upon OAPA treatment than the control group, while AO at all doses markedly decreased intracellular TG contents (Figure 3B). As illustrated in Figure 3C, AO maintained or enhanced the expression of *Ppara* and *Acox1* and significantly decreased the mRNA levels of *Fasn* in fat-loaded hepatocytes. Again, the gene expression of *Cpt1a* was not changed after AO treatment (Supplementary Figure S3B). Additionally, we examined the expression of targets involved in lipid metabolism at different time points or doses and found that AO significantly induced the activation of PPARα and ACOX1 after 1 h treatment and peaked at 4 h and decreased the levels of mature SREBP-1 and FASN at 1 h (Figures 3D,E and Supplementary Figure S3C, D). Furthermore, we determined the lipid-lowing effects of AO with the presence of overloaded fat. As expected, the protein level of lipid oxidation targets such as PPARα and ACOX1 were prominently increased and a lipogenic target like SREBP-1 maturation was decreased in response to various concentrations of AO when compared with the OAPA treatment alone (Figure 3F and Supplementary Figure S3E). Consistent with Figure 3E, AO also decreased the protein expression of FASN even in the presence of OAPA. These findings suggested that AO remarkedly inhibited lipid accumulation in hepatocytes.

### AO Rapidly Triggers Autophagy Flux in Hepatocytes

**FIGURE 4:**
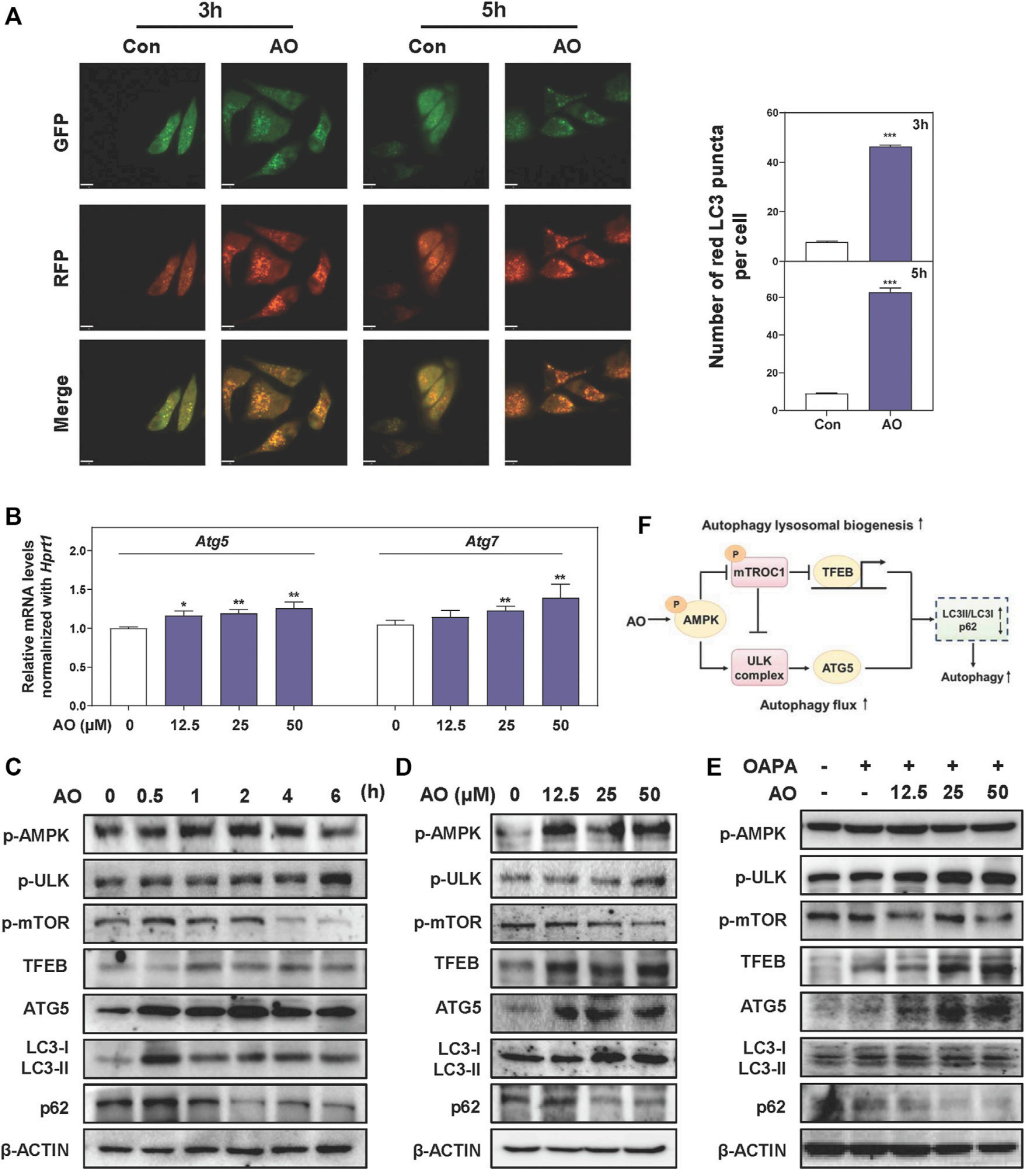
AO rapidly triggers autophagy flux in hepatocytes **(A)** MPHs were treated with AO (25 μM) for 3 and 5 h. The mean numbers of red puncta representing autolysosomes were plotted **(B)** Relative mRNA levels of *Atg5* and *Atg7* in MPHs were determined by qPCR and normalized using *Hprt1* as an internal control **(C)** MPHs were treated with AO (25 μM) at different time points **(D** and **E)** MPHs were treated with different concentrations of AO with or without OAPA **(C, D** and **E)** The protein levels of p-AMPK, p-ULK, p-mTOR, TFEB, ATG5, LC3-II/LC3-I and p62 in MPHs were determined by western blot using β-ACTIN as a loading control **(F)** Schematic diagram that linked AO with the regulation of autophagy. Statistical significance: *p < 0.05, **p < 0.01, ***p < 0.001, compared with control group (n = 3).

After observing that AO inhibited the lipid accumulation in MPHs, we further explored whether this lipid-lowing effect of AO was related to autophagy activation and was consistent with our *in vivo* findings. To examine whether AO itself affects the autophagic flux, we constructed an mRFP-GFP-LC3 reporter and transfected it into hepatocytes. As shown in Figure 4A, co-fluorescence staining of autolysosomes (red fluorescent puncta) and autophagosomes (yellow fluorescent puncta) exhibited that the number of red fluorescent puncta was sustainably increased by AO at 3 and 5 h, suggesting that AO effectively promoted autophagy flux in hepatocytes. As expected, AO significantly increased the mRNA levels of *Atg5* and *Atg7* in MPHs (Figure 4B). The oncogenic TFEB is considered to be the most important regulator of the transcription of genes responsible for the lysosomal-autophagy pathway (Settembre et al., 2011). As shown in Figure 4C, AO significantly increased the phosphorylation of AMPK and ULK at 0.5 h and peaked at 1 h, activated TFEB at 1 h and decreased the phosphorylation of mTOR, a downstream effector of AMPK, at 1 h and almost vanished at 4 h. Moreover, AO significantly resulted in the increase of LC3-II/I conversion and ATG5 and the decline of p62 in hepatocytes (Figure 4C and Supplementary Figure S4A). Consistent with the results above, the protein expressions of p-AMPK, p-ULK, TFEB, ATG5 and LC3-II were significantly increased while phosphorylated mTOR and p62 were markedly downregulated by AO treatment at different doses (Figure 4D and Supplementary Figure S4B). Given the strong influence of AO itself on autophagy activation, we next examined whether AO promoted autophagy exposure to high amounts of fatty acids. As depicted in Figure 4E and Supplementary Figure S4C, interestingly, OAPA exposure resulted in a compensatory increase of p-AMPK and TFEB but had no effect on ULK and mTOR phosphorylation, which were significantly enhanced by AO administration. As expected, AO significantly promoted the expression of autophagy-related proteins including ATG5 and LC3-II/LC3-I, while decreased the level of p62 compared with the OAPA group. These results suggested that AO rapidly triggered AMPK phosphorylation and promoted the autophagic flux in hepatocytes (Figure 4F).

### AO Alleviates Lipid Accumulation in Hepatocytes by Promoting AMPK-Mediated Protective Autophagy

**FIGURE 5:**
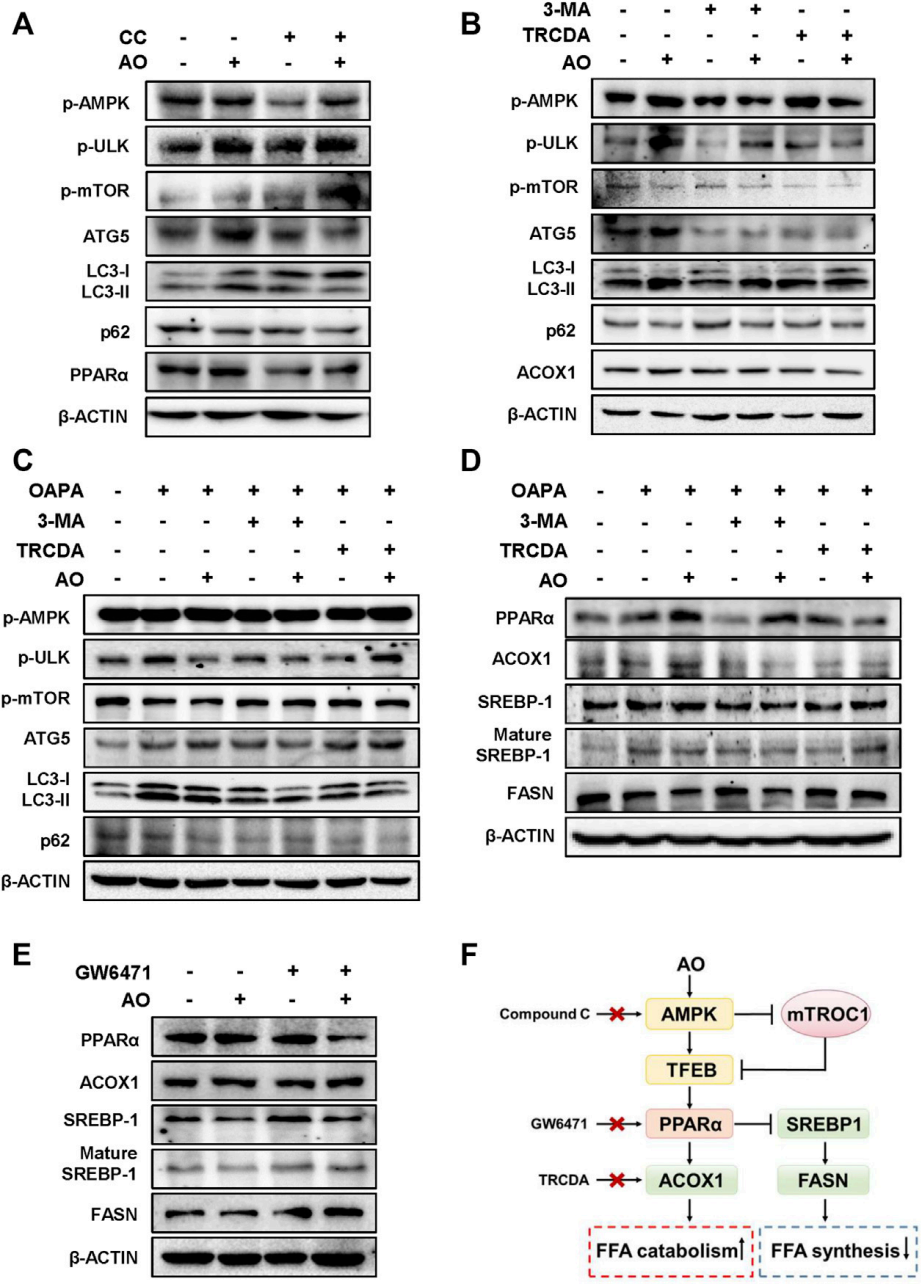
AO alleviates lipid accumulation in hepatocytes by promoting AMPK-mediated protective autophagy **(A)** MPHs were pre-treated with CC (10 μM) for 1.5 h and then administrated with AO (25 μM) for another 2 h **(B)** After pretreatment with 3-MA (5 mM) or TRCDA (10 μM) for 1.5 h, MPHs were administrated with AO (25 μM) for another 2 h **(C** and **D)** After pretreatment with 3-MA (5 mM) or TRCDA (10 μM) for 1.5 h, MPHs were administrated with AO (25 μM) for another 24 h with or without OAPA treatment **(E)** After pre-treated with GW6471 (10 μM) for 1.5 h, MPHs were administrated with AO (25 μM) for another 2 h **(A** to **E)** The protein levels of p-AMPK, p-ULK, p-mTOR, ATG5, LC3-II/LC3-I, p62 and PPARα, ACOX1, SREBP-1, mature SREBP-1 and FASN in MPHs were determined by western blot using β-ACTIN as a loading control **(F)** The pathway of AO in regulating FFA catabolism and synthesis.

We next applied different inhibitors of autophagy- or lipid metabolism-related targets and investigated their influences on the lipid-lowering effects of AO in hepatocytes. Firstly, the protein levels of p-AMPK and autophagy-related targets were examined after AO treatment with or without the presence of CC, a widely used AMPK inhibitor. In agreement with our anticipation, CC markedly prevented AO-induced AMPK phosphorylation, p-mTOR inhibition, PPARα induction and autophagic activity, as evidenced by decreased levels of ATG5, LC3-II and increased level of p62 (Figure 5A and Supplementary Figure S5A). Given the decisive role of AMPK in AO-induced autophagy activation, we next examined whether 3-MA, a specific inhibitor that blocks the formation of autophagosomes, had any effect on AMPK, autophagy- and lipid metabolism-related targets after AO treatment. As shown in Figure 5B and Supplementary Figure S5B, without affecting the phosphorylation of AMPK and ULK, 3-MA distinctly inhibited AO-induced autophagy flux, as evidenced by decreased LC3-II and ATG5 levels and increased p-mTOR and p62 levels. We also applied TRCDA, an inhibitor of Acox1 and found that AO failed to induce ACOX1 expression but still maintained the activation of phosphorylated AMPK and its downstream autophagy-related pathways with the presence of TRCDA (Figure 5B and Supplementary Figure S5B). Under the exposure to OAPA, 3-MA still inhibited the activation of autophagy caused by AO treatment, while TRCDA had no obvious effect on AO-induced autophagy activation, again, indicating that autophagy flux was triggered prior to ACOX1 induction (Figure 5C and Supplementary Figure S5C). After confirming that AO specifically activated autophagy under both normal and lipid-overload conditions, we next investigated whether the inhibition of autophagy had any effect on the lipid-reducing effect of AO in MPHs. As shown in Figure 5D and Supplementary Figure S5D, pretreated with 3-MA significantly blocked AO-induced upregulation of PPARα and ACOX1 and downregulation of SREBP-1 maturation, but exerted no effect on FASN expression. It was interesting to note that TRCDA slightly decreased PPARα and reversed the declined FASN level caused by AO, indicating complicated regulatory relationships between ACOX1 and FASN in hepatocytes. Both peroxisomal ACOX1 and mitochondrial CPT1α were regulated by the activation of PPARα-PGC1α transcription factor complex (Boeckmans et al., 2019). We then measured the changes of lipid metabolism-related targets after AO treatment with or without the presence of the PPARα inhibitor, GW6471. In accordance with our hypothesis, GW6471 significantly inhibited AO-induced upregulation of PPARα, causing distinct upregulation of mature SREBP-1 and FASN expression in hepatocytes (Figure 5E and Supplementary Figure S5E). Collectively, these results suggested that AO alleviated hepatic lipid accumulation and promoted FAO by promoting AMPK-mediated protective autophagy (Figure 5F).

### Inhibition of ACOX1 Blunted AO-Mediated Protective Effects Against Steatosis

**FIGURE 6:**
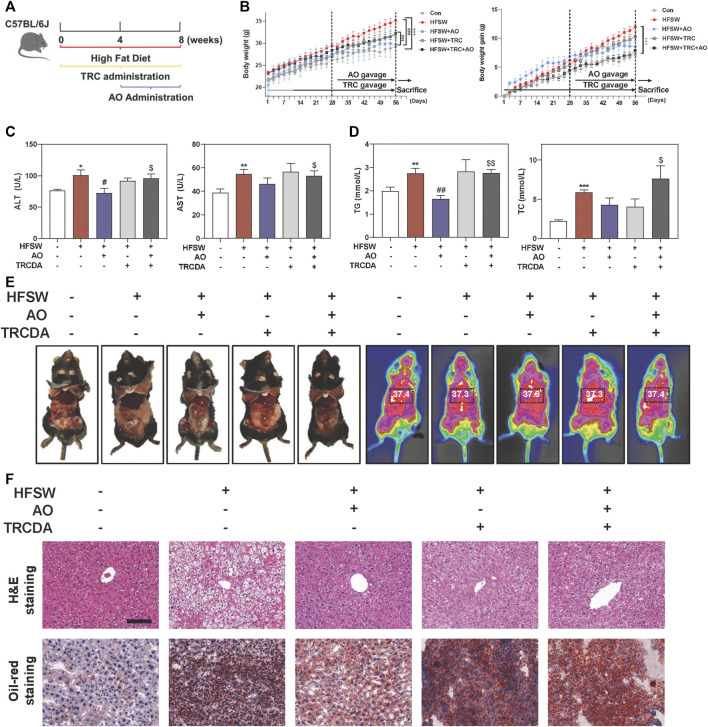
Inhibition of ACOX1 suppressed AO-mediated protective effects against NAFLD. Mice were fed chow diet or HFSW diet for 8 weeks, and administered with TRCDA (1 mg/kg) during the whole 8 weeks of HFSW diet followed by administration with AO (10 mg/kg) from Week 5 **(A)** Schematic diagram of *in vivo* experimental design **(B)** Body weight and Body weight gain **(C)** Serum ALT and AST levels **(D)** Serum TG and TC levels **(E)** Representative images of epididymal fat and thoracoabdominal temperature **(F)** Representative images of H&E and oil red O staining. Scale bar = 100 μm. Statistical significance: *p < 0.05, **p < 0.01, ***p < 0.001, compared with control group; ^#^ *p* < 0.05, ^##^ *p* < 0.01, compared with HFSW group; ^$^ *p* < 0.05, ^$$^ *p* < 0.01, compared with HFSW + AO group (n = 6).

We further focused on the common and paramount downstream target ACOX1 and identified whether AO-induced hepatoprotective effects depended on the ACOX1 activation. Based on our preliminary experiments, long-term administration of TRCDA (8 weeks) showed a better inhibitory effect on hepatic ACOX1 than short-term intervention (4 weeks) (**data not shown**). Therefore, mice were continuously treated with TRCDA accompanied with HFSW diet for 8 weeks and were co-administered with AO (10 mg/kg) during the last 4 weeks (Figure 6A). Although AO significantly inhibited the increase of body weight and weight gain induced by HFSW, TRCDA significantly elevated the body weight even in the presence of AO without affecting the amount of average calorie and water intake (Figure 6B and Supplementary Figure S6A). Serum biochemical examinations further showed that TRCDA significantly impaired the hepatoprotective effect of AO on fatty livers (Figure 6C). Moreover, the serum levels of TG and TC were distinctly reduced after AO treatment when compared with the HFSW group, which were significantly elevated by TRCDA (Figure 6D). It was interesting to note that additional administration of TRCDA obviously increased the weights of epididymal white adipose tissue and liver temperature when compared with the HFSW + AO group (Figure 6E). Furthermore, results of H&E, Oil-red staining and gross images of livers also showed that TRCDA remarkably increased the size and number of AO-declined lipid droplets (Figure 6F and Supplementary Figure S6B).

### Inhibition of ACOX1 Blocks the Lipid-Lowering Effect of AO *via* Disturbing Lipid Metabolism

**FIGURE 7:**
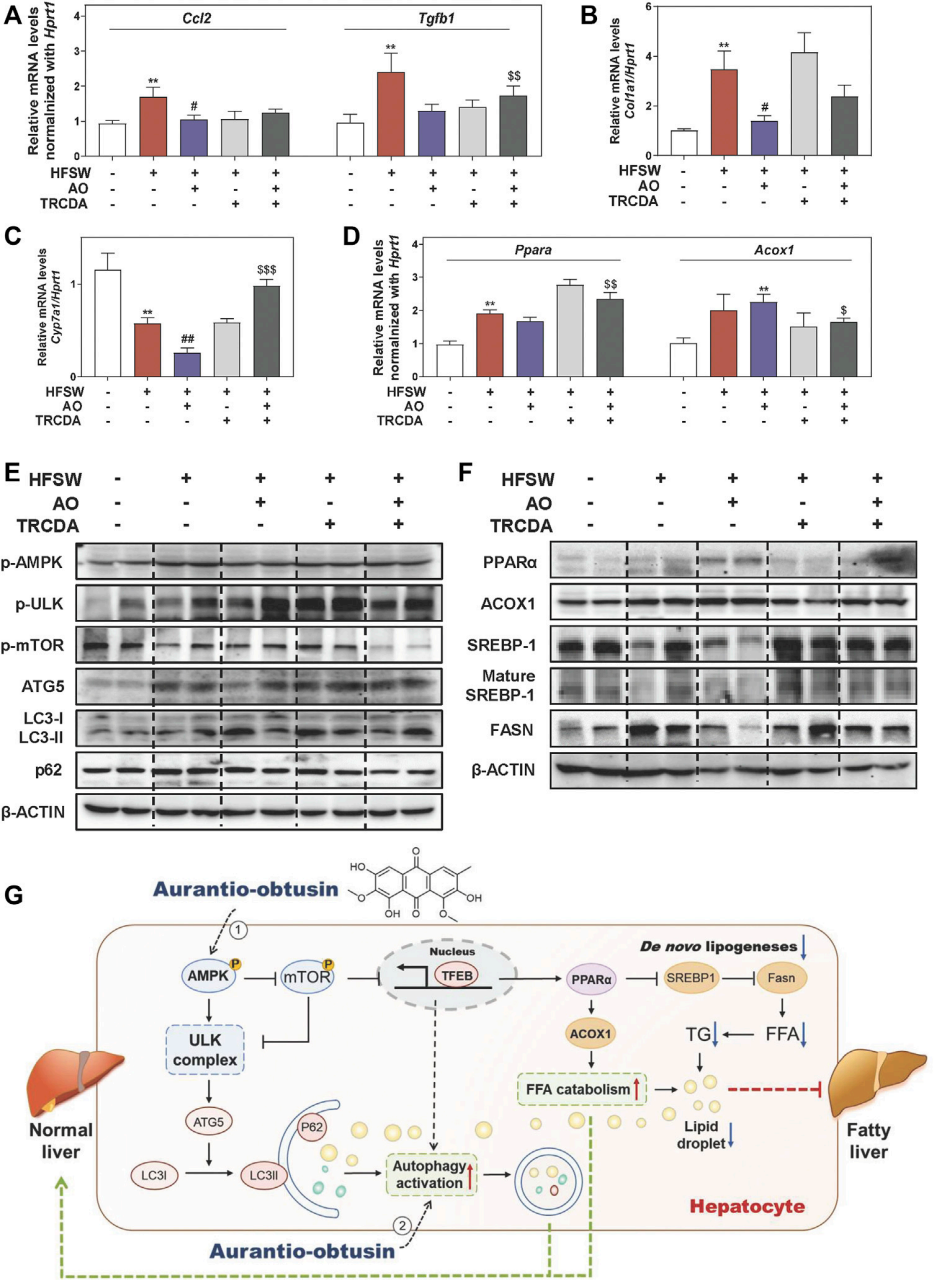
Inhibition of ACOX1 deteriorates the AO-ameliorated liver steatosis *via* accelerating liver inflammation and fibrosis and disturbing lipid metabolism. Relative mRNA levels of **(A)** *Ccl2* and *Tgfb1*, **(B)** *Col1a1*, **(C)** *Cyp7a1*, **(D)** *Ppara* and *Acox1* in liver tissues were determined by qPCR and normalized using *Hprt1* as an internal control. The protein levels of **(E)** p-AMPK, p-ULK, p-mTOR, ATG5, LC3-II/LC3-I, p62, **(F)** PPARα, ACOX1, SREBP-1, mature SREBP-1 and FASN in the liver were determined by western blot using β-ACTIN as a loading control, **(G)** Schematic diagram of the proposed mechanisms underlying the protective effects of AO on autophagy and lipid accumulation during NAFLD. Statistical significance: **p < 0.01, compared with control group; ^#^ *p* < 0.05, ^##^ *p* < 0.01, compared with HFSW group; ^$^ *p* < 0.05, ^$$^ *p* < 0.01, ^$$$^ *p* < 0.001, compared with HFSW + AO group (n = 6).

Previous studies reported that NAFLD mice caused by gene variation were in a proinflammatory and fibrotic state, accompanied by abnormal altered genes including ACOX1 (Dongiovanni et al., 2020). As shown in Figure 7A, B, and Supplementary Figure S6C, AO distinctly decreased the HFSW-induced levels of *Ccl2*, *Tgfb1* and *Col1a1* in livers, while TRCDA administration markedly reversed these changes and even increased the levels of *Fn1* and *Fasn* to a certain extent. We also noticed a significant elevation of the mRNA expression of *cyp7a1* in the HFSW + AO + TRCDA group when compared with the HFSW + AO group (Figure 7C). Considering the tight correlation between ACOX1 and PPARα, we next investigated whether the inhibition of ACOX1 had any influence on PPARα expression. As shown in Figure 7D, TRCDA markedly increased *Ppara* expression while decreased the level of *Acox1* induced by AO. Furthermore, the effects of ACOX1 inhibition on the autophagy signaling and lipid homeostasis were also investigated. As performed in Figure 7E and Supplementary Figure S6D, without affecting AMPK phosphorylation, TRCDA distinctly promoted AO-induced autophagy activation, as evidenced by a higher level of p-ULK, ATG5 and LC3-II/LC3-I and a lower level of p-mTOR and p62 compared to HFSW + AO group. These results indicated that the inhibition of ACOX1 not only didn’t block the AO-induced autophagy activation but even enhanced this process. In consistent with our results *in vitro*, TRCDA significantly reversed AO-suppressed FASN expression and SREBP-1 maturation in livers. Additionally, the expression of PPARα was slightly decreased in the HFSW + AO + TRCDA group when compared with the HFSW + AO group (Figure 7F and Supplementary Figure S6E). Collectively, our study provided critical evidence that the inhibition of ACOX1 aggravated HFSW-induced liver steatosis by promoting lipid *de novo* synthesis and inhibiting lipid consumption even in the case of autophagy activation (Figure 7G).

## Discussion

NAFLD affects about 20–30% of the adult population in developed countries and is inevitably becoming the next major health epidemic (Maya-Miles et al., 2021; Wegermann et al., 2021). Although a variety of therapeutic drugs curing NAFLD has been proposed, almost all of them have failed in the clinical trial, leading to an urgent requirement of novel therapeutic options. *Cassiae* semen is a prestigious and traditional drug that has been used for thousands of years in Asian history for treating various cardiovascular or digestive diseases. In the current study, we focused on AO, the main characteristic bioactive ingredient isolated from *Cassiae* semen, and found that AO significantly alleviated hepatic steatosis both in the HFSW-induced NAFLD mouse model and OAPA-treated lipid accumulation *in vitro* model, owing to the declined lipid *de novo* synthesis and elevated lipid consumption. Mechanistically, AO significantly activated the autophagy flux in hepatocytes through the phosphorylation of AMPK and the following activation of TFEB, which further improved the lipid metabolism by promoting the expression of PPARα and ACOX1 and inhibiting the maturation of SREBP-1 and downstream FASN expression (Figure 7G).

With the development of in-depth study of pathogenesis on NAFLD, the strong correlation between autophagy and hepatic lipid metabolism has been emphasized. Autophagy is responsible for the degradation of lipid droplets and critical for maintaining cellular energy homeostasis. Previous studies reported that the induction of hepatic autophagy effectively mitigated hepatic lipid accumulation in obese mice (L. Yang et al., 2010). Meanwhile, the increased production of unsaturated fatty acids was demonstrated to activate autophagy in multiple cell types (O'Rourke et al., 2013; Tu et al., 2014; B. Yang et al., 2020). In consistent with the *in vitro* experiments of oleate-induced autophagy activation by other studies (Singh et al., 2009), we found that HFSW feeding promoted the activation of autophagy to some extent by a compensatory mechanism. As shown in Figures 2, 4, AO efficiently improved hepatic lipid metabolism accompanied by increased autophagy flux and remarkable activation of ATG5. Previous studies have demonstrated that ATG5 could be activated under the phosphorylation of signal transducer and activator of transcription 3 (STAT3), the acetylation of p21 activated kinase 1 (PAK1) and induced hypoxia-inducible factor (Feng et al., 2021). Interestingly, we found that AO rapidly promoted the phosphorylation of STAT3 in hepatocytes (Supplementary Figure S7A). Therefore, we speculated that the activation of ATG5 induced by AO might be attributed to STAT3 phosphorylation, but detailed mechanisms remain to be identified in further studies. Furthermore, our results showed that AO markedly increased the ratio of LC3II/LC3I and decreased the level of p62. In addition, the function of AO on promoting autophagy and ameliorating lipid metabolism was suppressed by a classic early phase autophagy inhibitor, 3-MA. These data suggested that AO might participate in the regulation of the whole process of autophagy to modulate lipid metabolism and serve as a potent autophagy activator.

AMPK, an evolutionarily conserved serine/threonine-protein kinase, acts as an energy sensor and regulates a variety of metabolic processes (Paquette et al., 2021). Previous studies reported that AMPK directly promoted intracellular autophagy by phosphorylating the essential autophagy-related proteins including ULK and mTORC1 (Chen et al., 2020; Paquette et al., 2021; Wirth et al., 2013), and mTORC1 inhibited the interaction between AMPK and ULK1 (Loffler et al., 2011). In our study, we demonstrated that AO markedly increased the phosphorylation of AMPK and ULK and decreased the phosphorylation of mTOR (Figures 2, 4). Additionally, CC markedly blocked AO-induced upregulation of p-AMPK and p-ULK and downregulation of p-mTOR (Figure 5A). It has been previously reported that AMPK activated TFEB, a transcription factor that controls lysosomal biogenesis, and promoted the formation of autophagosomes (Settembre et al., 2011; Yoo et al., 2021). Here, we observed a significant increased level of TFEB under the challenges of various doses of AO in hepatocytes (Figure 4). Furthermore, Park *et al.* found that TFEB activation attenuated methionine choline-deficient diet-induced steatosis by increasing the FAO-related genes including *Ppara*, *Acox1* and *Cpt1a* (Park et al., 2020). Although there was no obvious change on *Cpt1a* level after AO treatment when compared with the control group, AO markedly increased the expression of PPARα and ACOX1, accompanied with increased TFEB level (Figure 3E). Taken together, our study provided critical evidence that AO activated autophagy and improved lipid metabolism by upregulating the expression of a series of autophagy-related proteins including AMPK, mTORC1, ULK as well as TFEB.

Previous studies reported that the induction of autophagy contributed to PPARα activation by promoting the degradation of NCoR1 (nuclear receptor co-repressor 1) (Saito et al., 2019). Additionally, it has also been reported that activated PPARα promoted the process of autophagy by up-regulating the expression of autophagy-related genes (Yu et al., 2020). A PPARα agonist, fenofibrate, was also found to activate AMPK pathway and promote TFEB nuclear translocation (Yoo et al., 2021). These findings suggest a complicated and interactive relationship between autophagy and PPARα. Interestingly, our results also identified that the inhibition of autophagy by 3-MA completely blocked AO-induced PPARα activation (Figure 5). In addition, previous studies have shown that AO increased the level of PPARγ in white tissues to improve obesity and insulin resistance (Guo et al., 2021), suggesting the potential activities of AO targeting PPAR family proteins. Similarly, we found that PPAR inhibitor GW6471 markedly eliminated AO-induced decreased level of FASN and mature SREBP-1 (Figure 5E), indicating AO exerted its lipid-lowering function in a PPARα-related manner.

ACOX1 has been well-characterized as the first step of peroxisomal β-oxidation and is responsible for the shortening of very long-chain fatty acids. Existing studies have suggested that the regulation of ACOX1 was largely under the control of activated PPARα (Misra and Reddy, 2014), while in the absence of ACOX1, unmetabolized ACOX1 substrates were reported to cause sustained activation of PPARα (Jia et al., 2014). Based on our *in vivo* and *in vitro* results, AO markedly stimulated lipid consumption and attenuated liver steatosis accompanied by the activation of PPARα and ACOX1 (Figures 2, 3). By using GW6471, we demonstrated that ACOX1 expression induced by AO was likely dependent on PPARα signaling. Whereas, enzymatic inhibition of ACOX1 by TRCDA slightly decreased AO-induced PPARα expression (Figures 5, 7). Meanwhile, the enhanced autophagy flux in response to ACOX1 inhibition was probably attributed to accumulated free fatty acids (Figure 7). Moreover, Anyuan He and his colleagues recently reported that hepatic Acox1 deficiency markedly led to lowered total cytosolic acetyl-CoA levels, resulting in impaired activation of mTORC1 (He et al., 2020a; He et al., 2020b), providing another explanation for enhanced autophagy after TRCDA administration. Interestingly, it is worth noting that the expression of FASN was also increased in liver-specific Acox1 knockout mice (He et al., 2020a), which was also consistent with our finding in Figure 7F, again, indicating the complex balance between lipid synthesis and lipid consumption in fatty liver.

## Conclusion

Insufficient therapeutic strategy for treating NAFLD remains a huge challenge of human public health. In the current study, we demonstrated that AO promoted autophagy flux and alleviated liver steatosis in an HFSW-fed NAFLD mouse model and OAPA-treated mouse primary hepatocytes by inducing the phosphorylation of AMPK and TFEB, which subsequently increased the expression of targets involved in lipid degradation and decreased the expression of targets involved in lipid biosynthesis. The inhibition of autophagy or genes responsible for lipid peroxidation markedly blocked AO-induced lipid-lowering and hepatoprotective effects. As illustrated in Figure 7G, our study not only provides insights into the complicated mechanisms underlying the anti-steatosis activities of AO but also offers vital evidence inspiring the development of AO structure-based innovative drug candidates for the treatment of NAFLD and related complications.

## Data Availability

The original contributions presented in the study are included in the article/Supplementary Material, further inquiries can be directed to the corresponding author.

## Abbreviations

AO, Aurantio-obtusin; ACOX1, acyl-CoA oxidase 1; AMPK, AMP-activated protein kinase; ATG5, autophagy-related protein 5; CC, compound C; CPT1α, carnitine palmitoyl transferase 1 alpha; FASN, fatty acid synthase; FFA, free fatty acids; HFSW, high-fat diet and glucose-fructose water; LC3, microtubule-associated protein 1 light chain 3; MPH, mouse primary hepatocyte; mTOR, mechanistic target of rapamycin; OAPA, oleic acid and palmitic acid; PPARα, peroxisome proliferator-activated receptor alpha; SREBP1, sterol regulatory element binding protein 1; TC, total cholesterol; TFEB, transcription factor EB; TG, triglycerides; TRCDA, 10,12-Tricosadiynoic acid; ULK, unc-51-like kinases.
