# Supplementary material for: Aurantio-Obtusin Attenuates Non-Alcoholic Fatty Liver Disease Through AMPK-Mediated Autophagy and Fatty Acid Oxidation Pathways
Source: Front Pharmacol. 2022 Jan 11;12:826628. doi: 10.3389/fphar.2021.826628 (PMC8787202; doi:10.3389/fphar.2021.826628)
Supplement: Supplementary file 1 [file DataSheet1.docx]

**
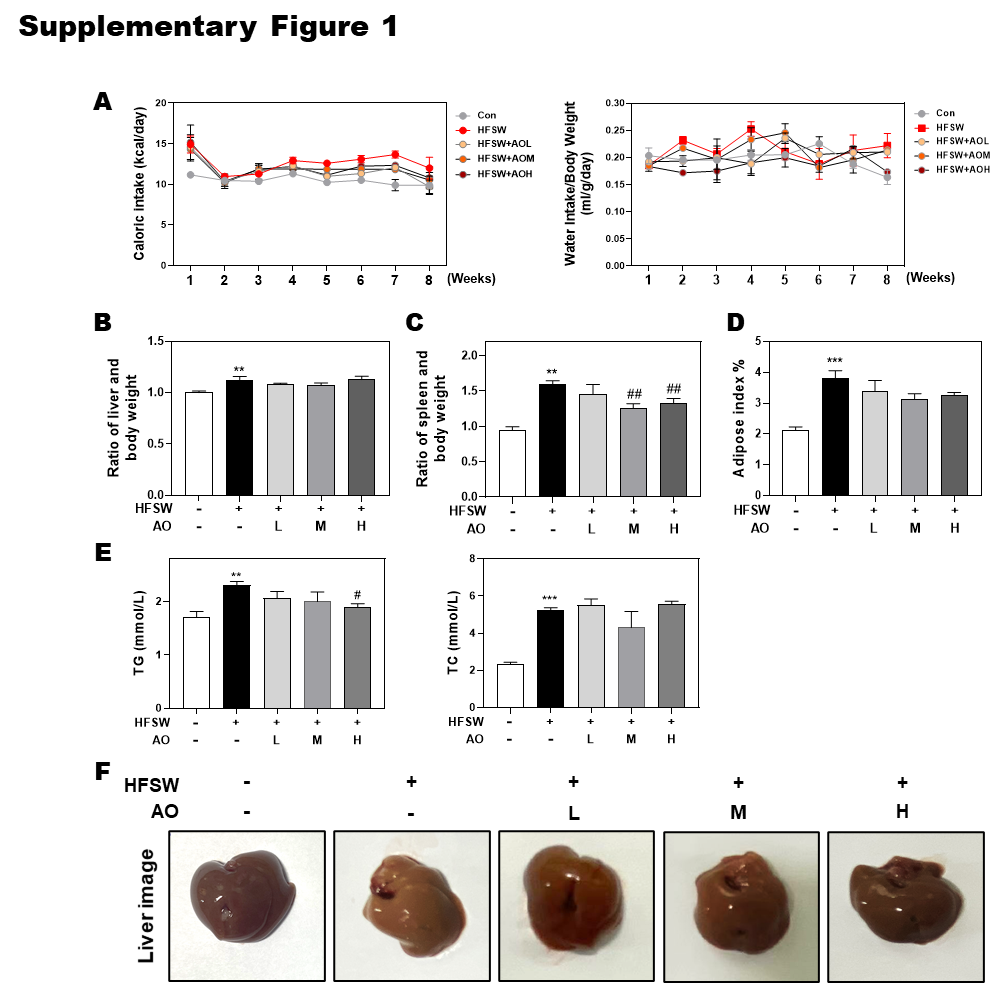
Supplementary figures and figure legends**

**Supplementary Figure 1. Effects of AO on HFSW-induced liver steatosis in mice. (A)** Caloric intake and water intake. **(B)** Ratio of liver and body weight. **(C)** Ratio of spleen and body weight. **(D)** Adipose index. **(E)** Serum TG and TC levels. **(F)** Gross liver images. Statistical significance: ***P*<0.01, ****P*<0.001, compared with control group; ^#^*P*<0.05, ^##^*P*<0.01, compared with HFSW group (n=6).

**
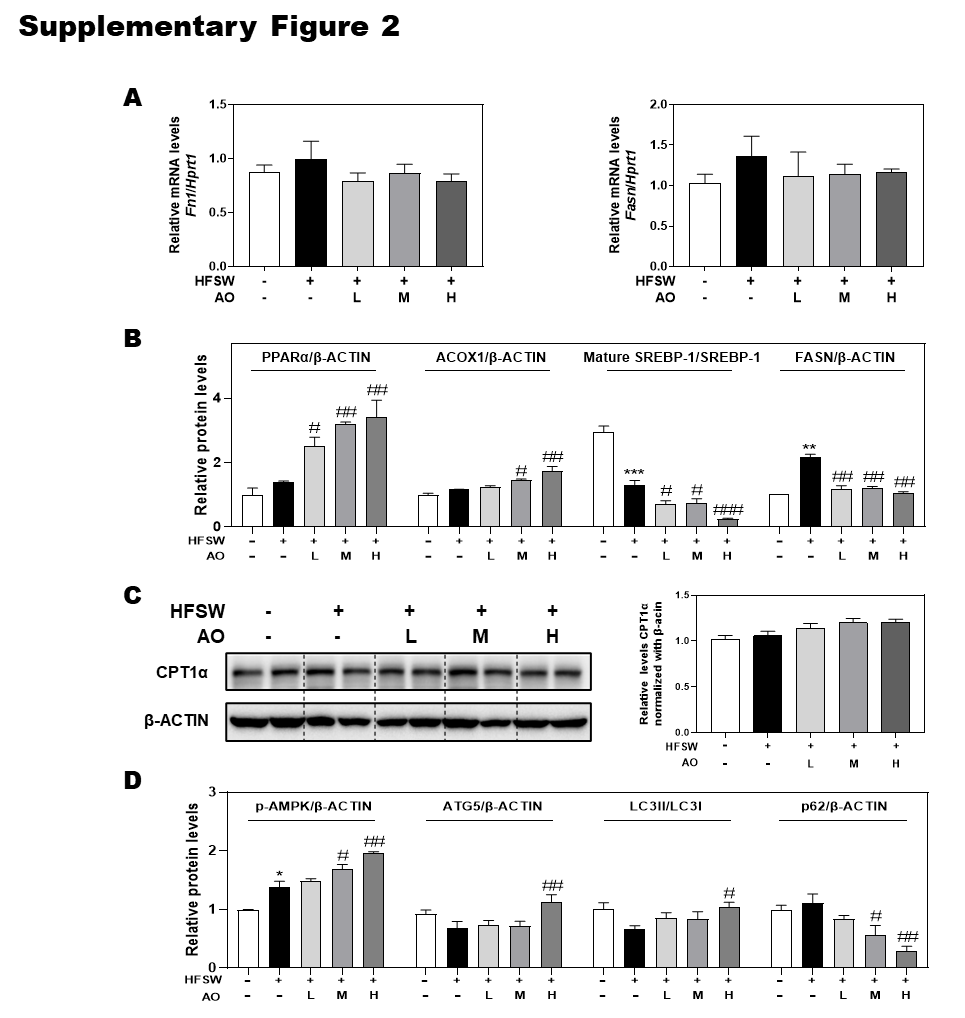
**

**Supplementary Figure 2. Effects of AO on *Fn1* and *Fasn* gene expressions and CPT1α protein expression and statistical analysis for western blot in HFSW-fed mice. (A)** Relative mRNA levels of *Fn1* and *Fasn* in liver tissues were determined by qPCR and normalized using *Hprt1* as an internal control. **(B** and **D)** The relative density of PPARα/β-ACTIN, ACOX1/β-ACTIN, mature SREBP-1/SREBP-1, FASN/β-ACTIN, p-AMPK/β-ACTIN, ATG5/β-ACTIN, LC3-II/LC3-1 and p62/β-ACTIN. **(C)** The protein level of CPT1α in the liver was determined by western blot using β-ACTIN as a loading control. Statistical significance: **P*<0.05, ***P*<0.01, ****P*<0.001, compared with control group; ^#^*P*<0.05, ^##^*P*<0.01, ^###^*P*<0.001, compared with HFSW group (n=6).


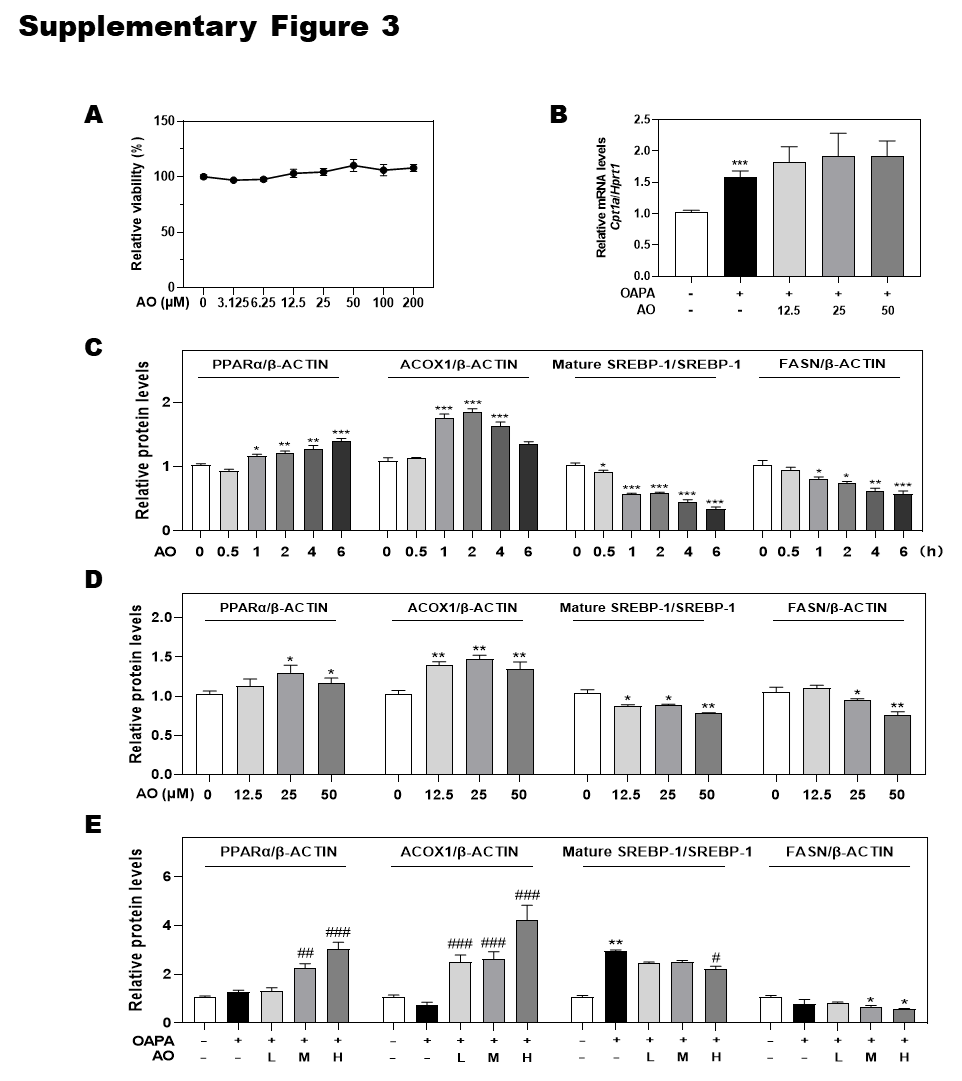


**Supplementary Figure 3. Effects of AO on cell viability and *Cpt1a* gene** **expression in OAPA-treated MPHs and statistical analysis for western blot in MPHs. (A)** A CCK-8 assay. **(B)** Relative mRNA level of *Cpt1a* in MPHs was determined by qPCR and normalized using *Hprt1* as an internal control. **(C**, **D** and **E)** The relative density of PPARα/β-ACTIN, ACOX1/β-ACTIN, mature SREBP-1/SREBP-1 and FASN/β-ACTIN. Statistical significance: **P*<0.05, ***P*<0.01, ****P*<0.001, compared with control group; ^#^*P*<0.05, ^##^*P*<0.01, ^###^*P*<0.001, compared with OAPA group (n=3).


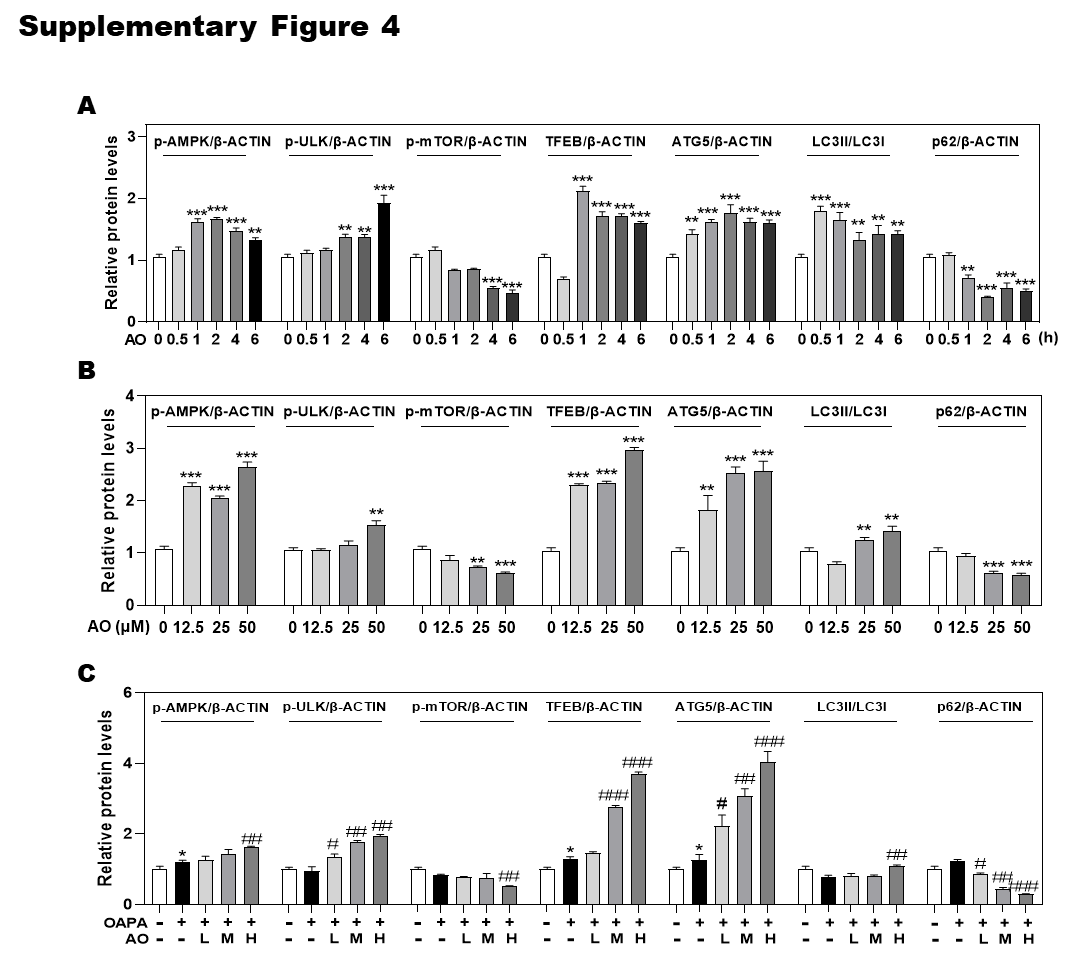


**Supplementary Figure 4. Statistical analysis for western blot in MPHs. (A, B** and **C)** The relative density of p-AMPK/β-ACTIN, p-ULK/β-ACTIN, p-mTOR/β-ACTIN, TFEB/β-ACTIN, ATG5/β-ACTIN, LC3-II/LC3-1 and p62/β-ACTIN. Statistical significance: **P*<0.05, ***P*<0.01, ****P*<0.001, compared with control group; ^#^*P*<0.05, ^##^*P*<0.01, ^###^*P*<0.001, compared with OAPA group (n=3).


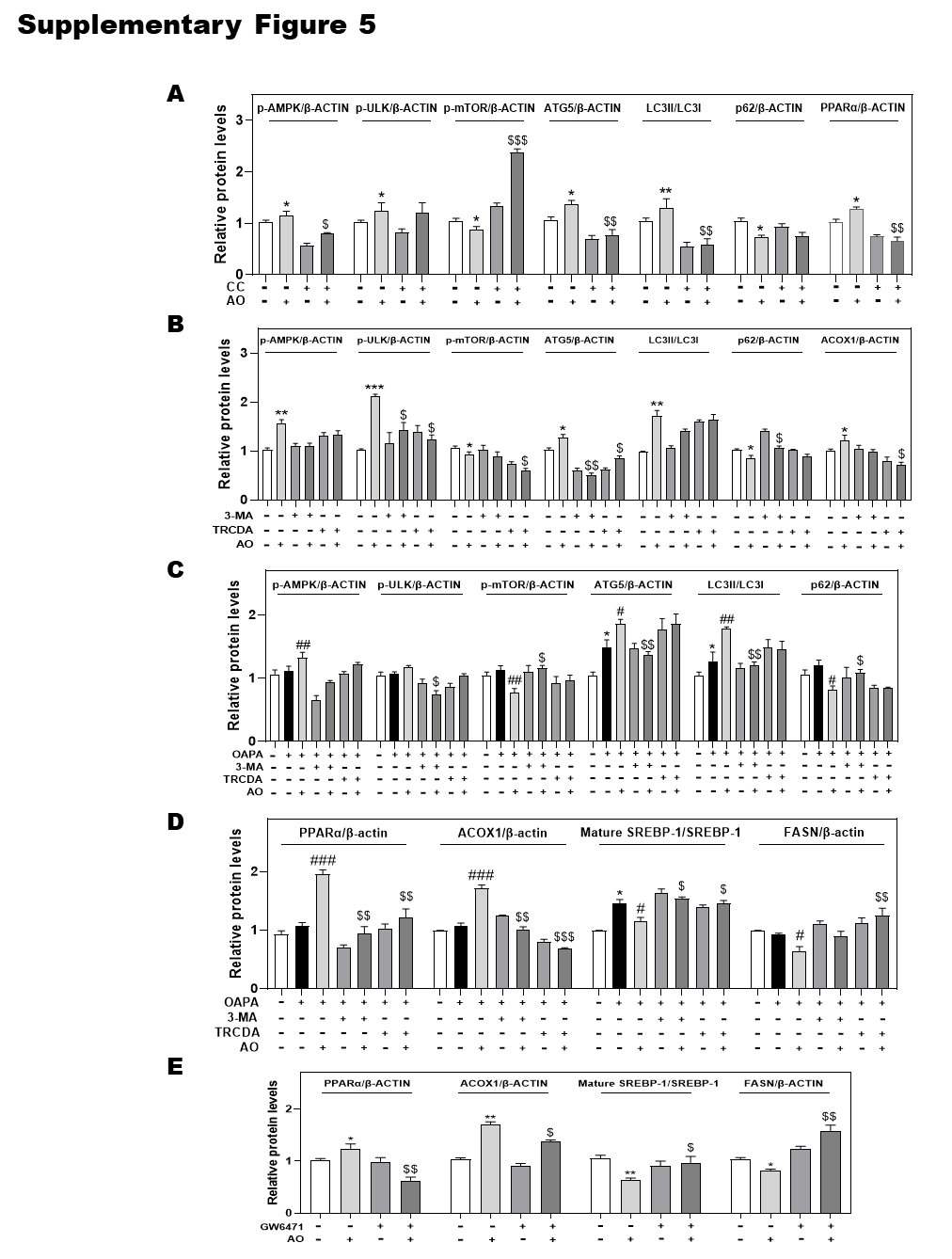


**Supplementary Figure 5. Statistical analysis for western blot in MPHs. (A** to **E)** The relative density of p-AMPK/β-ACTIN, p-ULK/β-ACTIN, p-mTOR/β-ACTIN, ATG5/β-ACTIN, LC3-II/LC3-I, p62/β-ACTIN, PPARα/β-ACTIN, ACOX1/β-ACTIN, mature SREBP-1/SREBP-1 and FASN/β-ACTIN. Statistical significance: **P*<0.05, ***P*<0.01, ****P*<0.001, compared with control group; ^#^*P*<0.05, ^##^*P*<0.01, ^###^*P*<0.001, compared with OAPA group; ^$^*P*<0.05, ^$$^*P*<0.01, ^$$$^*P*<0.001, compared with AO group with or without OAPA treatment (n=3).

**
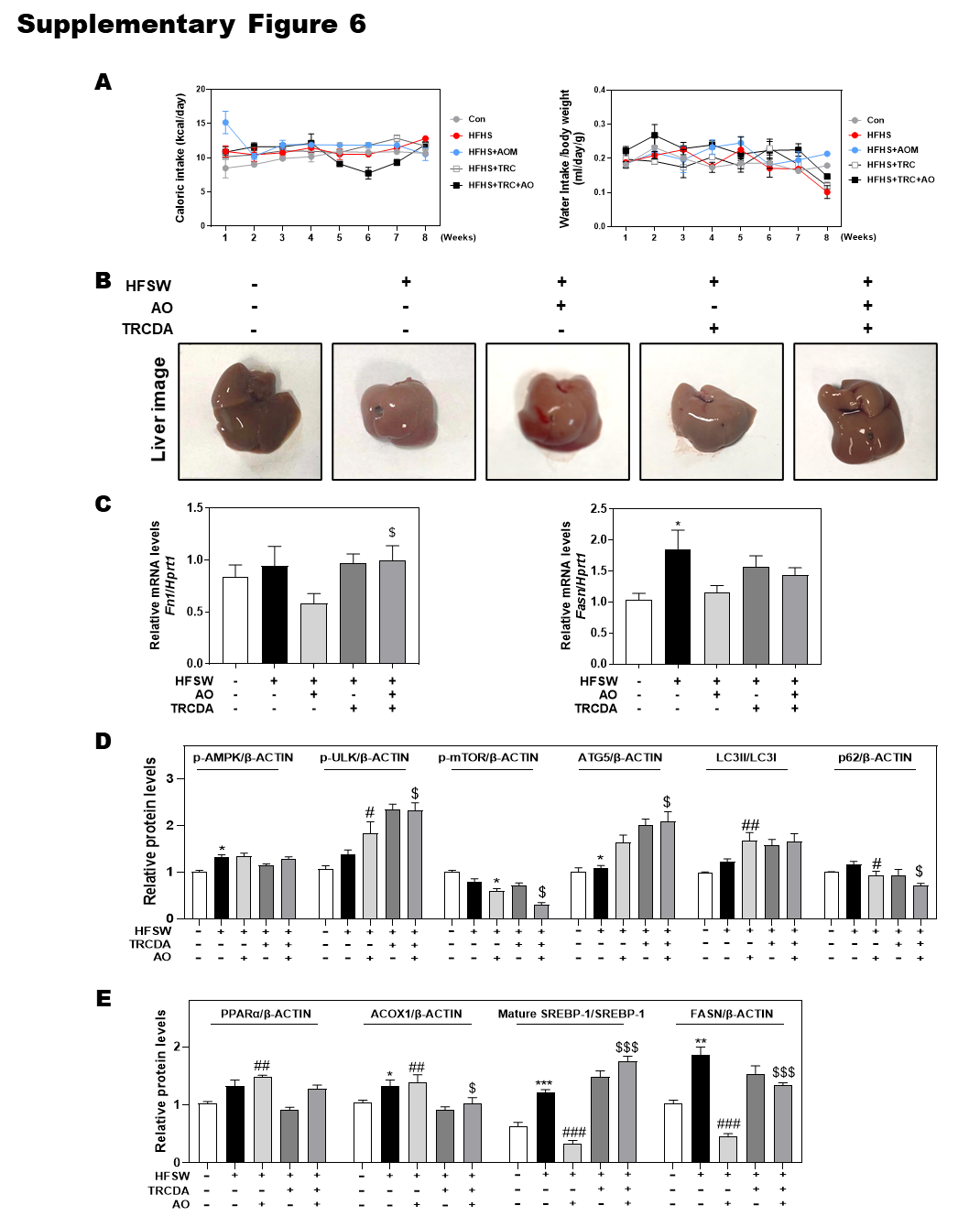
**

**Supplementary Figure 6. Effects of ACOX1 inhibition on AO-ameliorated liver steatosis and statistical analysis for western blot in mice. (A)** Caloric intake and water intake. **(B)** Gross liver images. **(C)** Relative mRNA levels of *Fn1* and *Fasn* in liver tissues were determined by qPCR and normalized using *Hprt1* as an internal control. **(D** and **E)** The relative density of p-AMPK/β-ACTIN, p-ULK/β-ACTIN, p-mTOR/β-ACTIN, ATG5/β-ACTIN, LC3-II/LC3-1 and p62/β-ACTIN, PPARα/β-ACTIN, ACOX1/β-ACTIN, mature SREBP-1/SREBP-1 and FASN/β-ACTIN. Statistical significance: **P*<0.05, ****P*<0.001, compared with control group; ^#^*P*<0.05, ^##^*P*<0.01, ^###^*P*<0.001, compared with HFSW group; ^$^*P*<0.05, ^$$$^*P*<0.001, compared with HFSW + AO group (n=6).

**
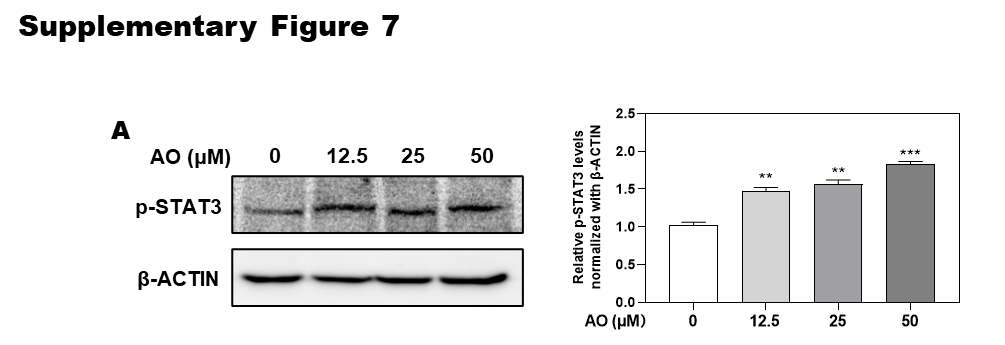
**

**Supplementary Figure 7. Effects of AO on the phosphorylation of STAT3 in OAPA-treated MPHs. (A)** The protein levels of p-STAT3 in the MPHs were determined by Western blot using β-ACTIN as a loading control. Statistical significance: ***P*<0.01, ****P*<0.001, compared with control group (n=3).

**Supplementary Table 1.** Primary antibodies for western blot experiment.

| **Antibody** | **Catalog number** | **Vendor** |
| --- | --- | --- |
| PPARα (H-2) | sc-398394 | Santa Cruz Biotechnology (Texas, USA) |
| SREBP1 | 14088-1-AP | Proteintech Group (Rosemont, USA) |
| FASN (G-11) | sc-48357 | Santa Cruz Biotechnology |
| ACOX1 | 10957-1-AP | Proteintech Group |
| CPT1*α* | 15184-1-AP | Proteintech Group |
| p-AMPK | Ab23875 | Abcam (Cambridge, USA) |
| p-ULK | 14202 | Cell Signaling Technology (Danvers, USA) |
| p-mTOR | 5536 | Cell Signaling Technology |
| TFEB | Sc-166736 | Santa Cruz Biotechnology |
| ATG5 | 10181-2-AP | Proteintech Group |
| LC3 | 14600-1-AP | Proteintech Group |
| p62 | 18420-1-AP | Proteintech Group |
| STAT3 | sc8019 | Santa Cruz Biotechnology |
| p-STAT3 | sc8059 | Santa Cruz Biotechnology |
| *β*-ACTIN | 4970s | Cell Signaling Technology |
